# Linear models for breeding values prediction in haplotype-assisted selection - an analysis of QTL-MAS Workshop 2011 Data

**DOI:** 10.1186/1753-6561-6-S2-S11

**Published:** 2012-05-21

**Authors:** Anna Mucha, Heliodor Wierzbicki

**Affiliations:** 1 Department of Genetics, Wrocław University of Environmental and Life Sciences, Kożuchowska 7, Wrocław 51-631, Poland

## Abstract

**Background:**

The aim of this study was to estimate haplotype effects and then to predict breeding values using linear models. The haplotype based analysis enables avoidance of loosing information due to linkage disequilibrium between single markers. There are also less explanatory variables in the linear model which makes the estimation more reliable.

**Methods:**

Different methods and criteria for marker and haplotype selection were considered. First, markers with MAF lower than 5% where excluded from the data set. Then, SNPs in complete linkage disequilibrium where selected. Next step was to construct haplotypes and to estimate their frequencies basing on selected SNPs. The haplotypes with a frequency lower than 1% were not considered in further analysis. Chosen haplotypes were used as the explanatory variables in the linear models for breeding values prediction. Linear models with fixed and random haplotype effects as well as animal model were tested.

**Results:**

The number of markers was limited to 1206, 1189, 1249, 1288 and 1167 for chromosome 1, 2, 3, 4 and 5, respectively due to MAF criterion. In total 409 subsets of SNPs with r^2^=1 were found. 1476 haplotypes with different lengths were inferred. The frequencies of 817 haplotypes were higher than 1% - 184 for the first chromosome, 172 for the second, 131 for the third, 146 for the forth and 184 haplotypes for the fifth chromosome. The haplotype effects estimated using random models were comparable and more precise in prediction for individuals with unknown phenotypes. A few haplotypes with large effects were found when their effects were defined as fixed in the linear model . The correlations of the predicted breeding values with true breeding values were not that high. This could be brought about by selection criteria imposed on the genotype data which led to substantial reduction of number of markers.

**Conclusions:**

Although not many markers were considered in the study, the results obtained show that the implemented approach can be considered as quite promising. The haplotype approach let to avoid high dimensional models as compared with single SNPs models.

## Background

Single Nucleotide Polymorphisms (SNPs) are the most widely used genetic markers for breeding value prediction [1]. Nonetheless, each SNP has relatively low content of genetic information. The haplotype approach gives a possibility to accumulate genetic information in haplotype blocks and to keep the Linkage Disequilibrium (LD) information in the statistical model [2]. Thus, the haplotype-assisted selection can be a very powerful tool in animal breeding [3].

## Methods

The QTL MAS 2011 simulated dataset was analysed to predict breeding values of individuals with known (2000 observations) and unknown (1000 observations) phenotypes. Genotype data were selected according to three criteria. Markers with Minor Allele Frequency (MAF) lower than 5% were excluded from the dataset. Then, LD between markers was measured using r^2^. SNPs in complete LD with at least one other SNP were picked out for further analysis. Basing on subsets of closely linked markers (MAF>5%, r^2^=1), haplotypes were constructed. Bayesian algorithm implemented in PHASE was used for haplotypes construction and for their frequencies estimation [4]. Haplotypes with population frequency lower than 1% were omitted in further analysis [5]. Inferred haplotype effects were estimated using statistical models for breeding values prediction. Four statistical models were considered. Fixed model (FM) handled haplotypes effects as fixed. The fitted model was the following: y = 1*_n_μ*_1_+*Xg*_1_+*e*_1_, where *y *is a vector of phenotypes, 1*_n _*is a vector of ones, *n *is number of known phenotypes, *μ*_1 _is an overall mean, *X *is a design matrix of haplotype effects, *g*_1 _is a vector of fixed haplotype effects, *e*_1 _is a vector of random residual effects and $e_{1}\sim N\left(0,\sigma_{e1}^{2}\right)$. Two random models (RM1 and RM2) treated haplotype effects as random. RM1 was the following: *y*=1*_n_μ*_2_+*Xg*_2_+*e*_2_, where *y*,1*_n_*, *n*, *μ*_2_, *X *are defined analogically as above, *g*_2 _is a vector of random haplotype effects and $g_{2}\sim N\left(0,\frac{\sigma_{g2}^{2}}{\#haplotypes}\right)$, *e*_2 _is a vector of random residual effects and $e_{2}\sim N\left(0,\sigma_{e2}^{2}\right)$. RM2 was the following: *y*=1*_n_μ*_3_+*Xg*_3_+*e*_3_, where *y*,1*_n_,n*, *μ*_3_, *X *are defined analogically as above, *g*_3 _is a vector of random haplotype effects and $g_{3}\sim N\left(0,\sigma_{g3}^{2}\frac{haplotypelength}{\#alleles}\right)$, *e*_3 _is a vector of random residual effects and $e_{3}\sim N\left(0,\sigma_{e3}^{2}\right)$. In RM1 the homogeneous variance whatever haplotype length, and in RM2 the heterogeneous variance depending of the haplotype length was assumed. Animal model (AM) was also fitted to the data to predict breeding values and to compare results obtained with previous models. AM was defined as follows: *y*=1*_n_μ*+*Zg*+*e*, where *y*,1*_n_*, *n,μ *are defined as in previous models, *Z *is a design matrix of random additive polygenic effects, *g *is a vector of random additive polygenic effects and $g\sim N\left(0,A\sigma_{g}^{2}\right)$, *A *is the numerator relationship matrix,*e *is a vector of random residual effects and $e\sim N\left(0,\sigma_{e}^{2}\right)$. The breeding values for individual *j *estimated using FM, RM1 and RM2 were defined as a sum of haplotype effects of the individual. The results of considered models were compared using the Pearson's correlation coefficients. All computations were performed using R-package.

## Results

### MAF and LD reduction

The results of MAF and LD reduction are shown in table 1. When MAF was used as a selection criterion the number of markers was limited from 1998 to 1206, 1189, 1249, 1288 and 1167 for chromosome 1, 2, 3, 4 and 5, respectively. LD between selected markers was investigated and the subsets of SNPs with r^2^=1 were allocated. 211 SNPs from the first chromosome were a base for construction of subsets of markers and inferring haplotypes. Analogically, 201 SNPs, 150 SNPs, 166 SNPs and 216 SNPs with r^2^=1 with at least one other marker were allocated for chromosome 2, 3, 4 and 5, respectively. Among selected SNPs different sizes of subsets were found. The sizes and numbers of SNPs subsets are shown in table 2. In total 409 subsets of SNPs were found. For example, 75 subsets consisted of 2 SNPs, 12 subsets consisted of 3 SNPs, 3 subsets consisted of 4 SNPs, 1 subset consisted of 5 SNPs and 1 subset consisted of 8 SNPs were obtained for the first chromosome. The results for remaining chromosomes can be read analogically from table 2.

**Table 1:** MAF and LD reduction

chromosome:	1	2	3	4	5
all markers	1998	1998	1998	1998	1998

markers with MAF>0.05	1206	1189	1249	1288	1167

markers with MAF>0.05 and r^2^=1	211	201	150	166	216

**Table 2:** Subsets of SNPs after MAF and LD reduction

chromosome	subset of SNPs							
	
	all	2-SNP	3-SNP	4-SNP	5-SNP	6-SNP	7-SNP	8-SNP
1	92	75	12	3	1	-	-	1

2	87	67	16	3	-	-	1	-

3	65	52	8	3	2	-	-	-

4	73	57	12	4	-	-	-	-

5	92	71	14	4	2	1	-	-

TOTAL	409	322	62	17	5	1	1	1

### Reduction by haplotype frequencies

A total of 1476 haplotypes with different lengths were inferred - 328 for the first chromosome, 309 for the second, 240 for the third, 262 for the forth and 337 haplotypes for the fifth chromosome. The frequencies of 817 haplotypes were higher than 1% - 184 for the first chromosome, 172 for the second, 131 for the third, 146 for the forth and 184 haplotypes for the fifth chromosome (table 3). Among haplotypes with frequency higher than 1%, there were 644 haplotypes consisted of 2 alleles, 123 haplotypes consisted of 3 alleles, 34 haplotypes consisted of 4 alleles, 10 haplotypes consisted of 5 alleles, 2 haplotypes consisted of 6 alleles, 2 haplotypes consisted of 7 alleles and 2 haplotypes consisted of 8 alleles (table 3).

**Table 3:** Number of haplotypes according to chromosome, haplotype length and frequency

Haplotype length	subset	chromosome					TOTAL
			
		1	2	3	4	5	
all	all	328	309	240	262	337	1476
	
	freq>1%	184	172	131	146	184	817

2	all	232	215	173	187	235	1042
	
	freq>1%	150	134	104	114	142	644

3	all	56	70	35	49	44	254
	
	freq>1%	24	30	17	24	28	123

4	all	18	14	16	26	24	98
	
	freq>1%	6	6	6	8	8	34

5	all	8	-	16	-	22	46
	
	freq>1%	2	-	4	-	4	10

6	all	-	-	-	-	12	12
	
	freq>1%	-	-	-	-	2	2

7	all	-	10	-	-	-	10
	
	freq>1%	-	2	-	-	-	2

8	all	14	-	-	-	-	14
	
	freq>1%	2	-	-	-	-	2

### Breeding values prediction

**Figure 1:**
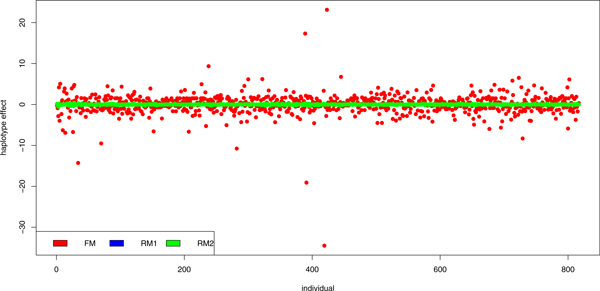
**Haplotype effects**. Figure shows the scale of haplotype effects estimated using fixed model (FM), the first random model (RM1) and the second random model (RM2).

The constructed haplotypes were used for breeding values prediction. First, the haplotype effects estimated using FM, RM1 and RM2 were investigated. The results of FM and RM1 estimation are shown in Figure 1. The haplotype effects estimated using RM1 and RM2 were highly comparable. The correlation between them was 0.9607. The FM results differed markedly as compared with the random models results. The correlation between haplotype effects estimated using FM and RM1 was 0.1737, whereas the correlation between haplotype effects estimated using FM and RM2 was 0.1656.

**Figure 2:**
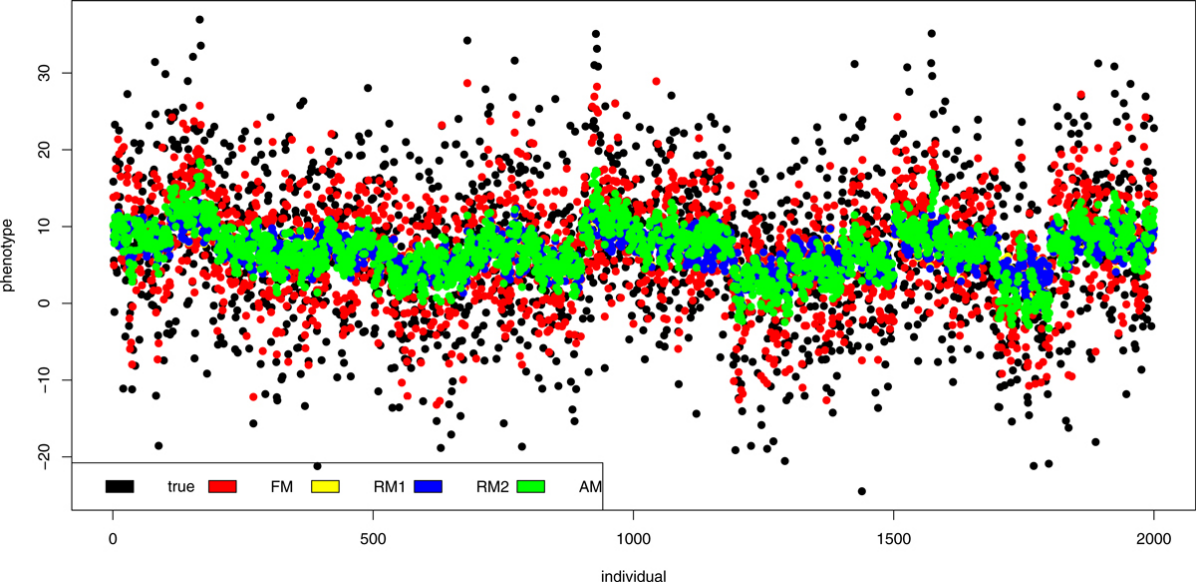
**True and predicted phenotypes (individuals with known phenotype)**. Figure shows true and predicted values of phenotypes for individuals with known phenotype. The predictors were calculated using the fixed model (FM), the first random model (RM1), the second random model (RM2) and the animal model (AM).

**Figure 3:**
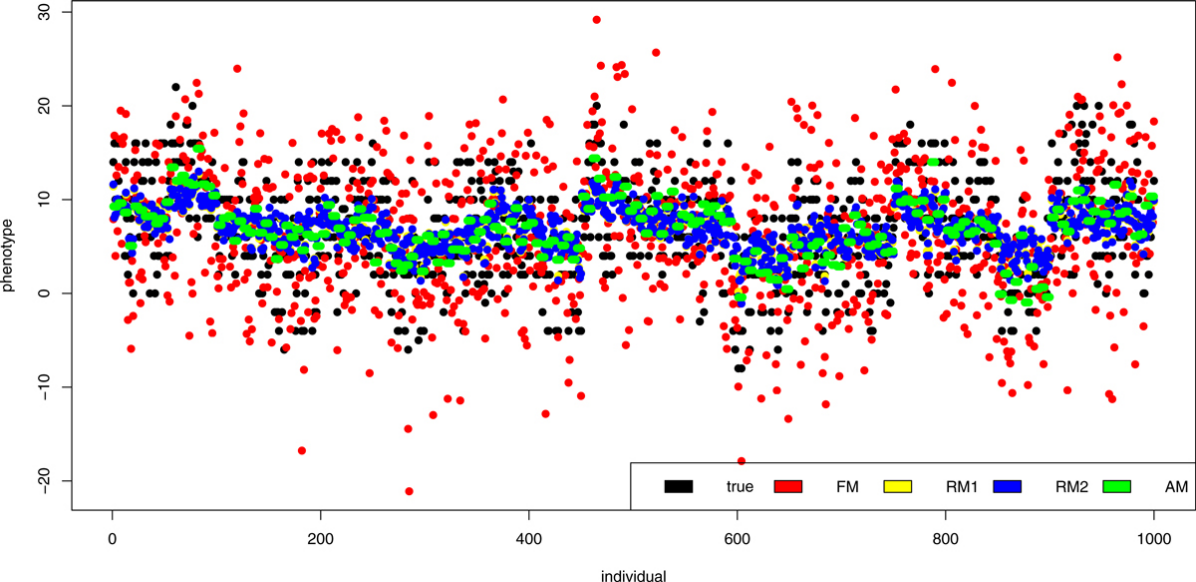
**True and predicted phenotypes (individuals with unknown phenotype)**. Figure shows true and predicted values of phenotypes for individuals with unknown phenotype. The predictors were calculated using the fixed model (FM), the first random model (RM1), the second random model (RM2) and the animal model (AM).

All models were used for breeding values and thereafter for phenotype values prediction, especially for the individuals with unknown phenotype (all phenotypes were published after QTL MAS Workshop 2011). The results of phenotypes prediction and the true values for individuals with known phenotypes are shown in Figure 2. The results of FM and AM were closer to the true values than other results. The correlation between true phenotypes and the FM results was 0.7145, whereas between true phenotypes and the AM results was 0.7315. The phenotypes predicted using random models (RM1 and RM2) were highly comparable (with the correlation of 0.9974 between them), but less correlated with true values (0.4872 and 0.4911, respectively), than FM and AM results. These and remaining correlations were statistically significant (p < 0.05) and are shown in table 4. The results of phenotypes prediction and the true values for individuals with unknown phenotypes are shown in Figure 3, which shows that the results of RM1 and RM2 were more precise than the other ones. The correlations between phenotypes predicted with these models and true values were 0.7043 and 0.7052, respectively. These predictors were also very similar (correlation 0.9972). In case of unknown phenotypes FM gave less precise results. The correlation with the true value was 0.4873. The AM predictors were correlated with true values at 0.6081. These and remaining correlations were statistically significant (p < 0.05) and are shown in table 4.

**Table 4:** Correlations between true and predicted phenotypes

MODEL	true	FM	RM1	RM2	AM
true	1	0.4873 (0.4385, 0.5331)	0.7043 (0.6716, 0.7342)	0.7052 (0.6726, 0.7351)	0.6081 (0.5675, 0.6458)

FM	0.7145 (0.6924, 0.7353)	1	0.5396 (0.4942, 0.5821)	0.5443 (0.4991, 0.5865)	0.4306 (0.3787, 0.4797)

RM1	0.4872 (0.4531, 0.5200)	0.6819 (0.6577, 0.7047)	1	0.9972 (0.9968, 0.9975)	0.7631 (0.7360, 0.7879

RM2	0.4911 (0.4571, 0.5236)	0.6873 (0.6635, 0.7097)	0.9974 (0.9972, 0.9976)	1	0.7616 (0.7342, 0.7864)

AM	0.7315 (0.7105, 0.7513)	0.7174 (0.6955, 0.7381)	0.7921 (0.7751, 0.8078)	0.7939 (0.7771, 0.8096)	1

The correlations between true and predicted phenotypes for individuals with known phenotype (below diagonal). The correlations between true and predicted phenotypes for individuals with unknown phenotype (above diagonal). 95% confidence intervals for correlations are shown in brackets.

## Discussion

The MAF and LD reduction results were comparable and there were not substantial differences between chromosomes. The haplotypes consisted of 2 alleles were predominant. The longest haplotype length was 8 alleles. The longer haplotype, the lower was its frequency and the less haplotypes fulfilled the threshold of 1%. A few haplotypes with large effects were found using the fixed model. The negligible differences between results obtained using RM1 and RM2 were probably caused by small disparities between haplotype lengths (from 2 to 8 alleles). Regardless of heterogeneous (RM2) or homogeneous (RM1) variance assumption, the breeding values prediction results were comparable. FM and AM gave better results for the individuals with known phenotypes, whereas RM1 and RM2 were more precise in prediction for individuals with unknown phenotypes. The correlations of the predicted breeding values with true breeding values were not high and ranged from 0.4872 to 0.7315. This could be brought about by selection criteria imposed on the genotype data which led to substantial reduction of number of markers.

## Conclusions

Although not many markers were considered in the study (outcome of complete LD as a marker selection criterion), the results obtained show that the implemented approach can be considered as quite promising. The random models (RM1 and RM2) gave highly comparable results, more precise for individuals with unknown phenotypes. The haplotype approach let to avoid high dimensional models as compared with single SNPs models.

## List of abbreviations used

SNP: Single Nucleotide Polymorphisms; LD: Linkage Disequilibrium; MAF: Minor Allele Frequency; FM: Fixed model; RM: Random models; AM: Animal model.

## Competing interests

The authors declare that they have no competing interests.
